# Transmissibility and severity of COVID‐19 in a humanitarian setting: First few X investigation of cases and contacts in Juba, South Sudan, 2020

**DOI:** 10.1111/irv.13200

**Published:** 2023-11-17

**Authors:** Richard Lino Loro Lako, Niamh Meagher, Joseph Francis Wamala, John Ndyahikayo, Ayesheshem Ademe Tegegne, Olushayo Oluseun Olu, David J. Price, Soatiana Rajatonirina, Elise Farley, Joseph Chukwudi Okeibunor, Valerie Ann Mize

**Affiliations:** ^1^ Government of the Republic of South Sudan Ministry of Health Juba South Sudan; ^2^ Department of Infectious Diseases The University of Melbourne, at The Peter Doherty Institute for Infection and Immunity Melbourne Australia; ^3^ World Health Organization Juba South Sudan; ^4^ Centre for Epidemiology & Biostatistics, Melbourne School of Population & Global Health The University of Melbourne Melbourne Australia; ^5^ World Health Organization, AFRO Brazzaville Congo

**Keywords:** COVID‐19, first few X, South Sudan, transmission investigation

## Abstract

**Background:**

The first few ‘X’ (FFX) studies provide evidence to guide public health decision‐making and resource allocation. The adapted WHO Unity FFX protocol for COVID‐19 was implemented to gain an understanding of the clinical, epidemiological, virological and household transmission dynamics of the first cases of COVID‐19 infection detected in Juba, South Sudan.

**Methods:**

Laboratory‐confirmed COVID‐19 cases were identified through the national surveillance system, and an initial visit was conducted with eligible cases to identify all close contacts. Consenting cases and close contacts were enrolled between June 2020 and December 2020. Demographic, clinical information and biological samples were taken at enrollment and 14–21 days post‐enrollment for all participants.

**Results:**

Twenty‐nine primary cases and 82 contacts were included in the analyses. Most primary cases (*n* = 23/29, 79.3%) and contacts (*n* = 61/82, 74.4%) were male. Many primary cases (*n* = 18/29, 62.1%) and contacts (*n* = 51/82, 62.2%) were seropositive for SARS‐CoV‐2 at baseline. The secondary attack rate among susceptible contacts was 12.9% (4/31; 95% CI: 4.9%–29.7%). All secondary cases and most (72%) primary cases were asymptomatic. Reported symptoms included coughing (*n* = 6/29, 20.7%), fever or history of fever (*n* = 4/29, 13.8%), headache (*n* = 3/29, 10.3%) and shortness of breath (*n* = 3/29, 10.3%). Of 38 cases, two were hospitalised (5.3%) and one died (2.6%).

**Conclusions:**

These findings were used to develop the South Sudanese Ministry of Health surveillance and contract tracing protocols, informing local COVID‐19 case definitions, follow‐up protocols and data management systems. This investigation demonstrates that rapid FFX implementation is critical in understanding the emerging disease and informing response priorities.

## 1 INTRODUCTION

Upon the emergence of a novel pathogen, there is substantial uncertainty over the key epidemiological, clinical and virological characteristics of the pathogen, particularly its ability to spread in the human population (transmissibility) and its virulence (infection severity). The first few 'X’ (FFX) study design facilitates a better understanding of these characteristics through prospective follow‐up of cases and their close contacts in the early stages of an epidemic or pandemic. Early implementation of FFX investigations provides timely, setting‐specific evidence that can be used to guide public health decision‐making and resource allocation,^1^ especially in times when there is limited data to inform these policies. This was the situation for coronavirus disease 2019 (COVID‐19), which was declared a pandemic in March 2020, and during the early stages of the outbreak, the infectious period, the route of transmission, the full range of disease presentation and the viral dynamics were largely unknown.^2^

In South Sudan, the first COVID‐19 case was reported on 5 April 2020.^3^ Three main peaks in cases occurred from April to June 2020, December to March 2021, and December 2021 to January 2022.^4^ As of 10 October 2022, there had been 17,780 confirmed cases of COVID‐19 and 138 deaths reported to the World Health Organization (WHO).^5^ This case fatality rate (0.8%) is lower than reported elsewhere in the region,^6^, ^7^ however, these epidemiological indicators need to be interpreted bearing in mind the policy, funding and strategy decisions in South Sudan at this time.^8^ Chronic underdevelopment due to prolonged civil war and several major civil conflicts have left South Sudan with one of the weakest health systems in the world.^7^

Lockdown measures, including school closures and restriction on mass gatherings and commercial flights, were implemented in South Sudan in March 2020, approximately 3 weeks before confirmation of the first cases,^7^ and these restrictions were gradually eased in May 2020.^8^ Despite the early interventions, weaknesses in the health system greatly impacted the response to COVID‐19. Challenges included limited testing availability and capacity, logistical and financial challenges and overburdened and understaffed health facilities. These challenges undoubtedly changed the course of the outbreak in the country and impacted reporting case and mortality figures.^8^

Given limitations in surveillance, there was a need to better understand the transmission dynamics and clinical course of COVID‐19 in a humanitarian setting such as South Sudan. For this reason, in June 2020, the Ministry of Health of South Sudan began recruitment of COVID‐19 cases and their close contacts in Juba, South Sudan, for an FFX investigation using an approach adapted from the WHO Unity FFX investigation protocol.^1^ The overall aim was to gain an early understanding of key clinical, epidemiological and virological characteristics of COVID‐19 to help inform the development of public health guidance, manage cases and reduce the potential spread and impact of infection in the country.

## 2 METHODS

### 2.1 Design and setting

We conducted a prospective, case‐ascertained investigation of identified close contacts of laboratory‐confirmed COVID‐19 cases living in Juba, South Sudan, using the WHO Unity FFX investigation protocol, which was adapted for the local context.^9^

### 2.2 Participants

All individuals who received a positive reverse transcription polymerase chain reaction (RT‐PCR) test for COVID‐19 (i.e., laboratory‐confirmed COVID‐19) reported to the Ministry of Health of South Sudan were considered eligible for enrollment as primary cases. The Ministry was notified of eligible COVID‐19 cases via a national COVID‐19 hotline hosted in the Public Health Operations Centre. Cases were identified through travel screening or through expanded surveillance practices for influenza‐like illness and severe acute respiratory illness in sentinel sites and both public and private health facilities.

All known close contacts in an index case were eligible for inclusion. All close contacts were recorded, and the first two to four contacts who were eligible and willing to participate were enrolled. Cases and contacts were excluded if they lived outside of Juba, if they had previously been enrolled, if they were not able to be contacted or if they declined to take part in the investigation. Eligible index cases who could not identify or disclose their contacts were also excluded from the investigation.

### 2.3 Data collection

SARS‐CoV‐2 test results were reported to the Ministry of Health daily to facilitate case investigation and contact tracing by dedicated COVID‐19 rapid response teams. Where eligible cases consented to be included in the investigation, the rapid response teams conducted an initial visit with the primary case to identify all close contacts, to collect relevant sociodemographic and clinical information and to collect biological samples (nasopharyngeal and oropharyngeal swabs and blood samples).

Enrolled contacts were then visited to gather clinical information and biological samples (nasopharyngeal and oropharyngeal swabs and blood samples). Contacts were also asked to keep symptom diaries until subsequent follow‐up. Both cases and contacts were followed up with a second visit 14–21 days after enrollment. At this visit, clinical information and biological samples were taken from cases (nasopharyngeal and oropharyngeal swabs and blood samples) and contacts (oropharyngeal swabs and blood samples). Symptoms, comorbidities and use of healthcare facilities were self‐reported by all participants; where this data was missing, it was assumed that the participant did not have the symptom or co‐morbidity and did not access healthcare.

All information on primary cases and their respective contacts was captured on FFX case and contact questionnaires. The data was entered into the GoData software platform^10^ by trained research team members.

### 2.4 Laboratory testing

National protocols adapted from WHO guidelines were followed for specimen collection, transportation and testing.^11^ WANTAI SARS‐CoV‐2 antibody ELISA tests were used for serology testing, and RT‐PCR tests were conducted on Rotor‐Gene Q and BioRad CFX96 machines. All tests were conducted in Juba by the Ministry of Health's National Public Health Laboratory team.

### 2.5 Definitions

The classification of participants as primary, co‐primary or secondary cases was based on the timing of positive COVID‐19 tests and symptom onset dates, as per the WHO Unity FFX investigation protocol.^9^ The definitions used in the study are outlined in Table 1.

**TABLE 1:** Definitions used to classify cases in the investigation.

Term	Definition
Index case	The first identified laboratory‐confirmed COVID‐19 case that triggered recruitment into the investigation.
Primary case	The laboratory‐confirmed COVID‐19 case with the first evidence of infection (at least 24 h before the symptom onset of any other positive cluster members).
Co‐primary case(s)	The terminology was used when it was unclear who was the primary case—that is, when the index case and one or more contacts tested positive within 24 h of each other, and the primary case was not able to be identified using the earliest symptom onset date.
Secondary case	Contacts who had either: A negative RT‐PCR COVID‐19 test result at baseline, and a positive COVID‐19 RT‐PCR result on or before Day 21, or; Negative serology for COVID‐19 at baseline, and positive serology for COVID‐19 at follow‐up, 14–21 days later.

A contact was defined as a person who experienced any one of the following exposures during the period of 2 days before and 14 days after the positive test or onset of symptoms of an index case:

1. Face‐to‐face contact with an index case within 1 m and for more than 15 min.
2. Direct physical contact with an index case.
3. Direct care for a patient who is an index case without using proper personal protective equipment; or.
4. Other situations as indicated by local risk assessments.

A household contact was defined as a person who was exposed to the index case in the household with a long duration of exposure (i.e., days in length). Contacts described as being a neighbour, co‐worker or workmate are excluded from this definition.

The infection secondary attack rate (SAR) was defined as the proportion of contacts who had a laboratory‐confirmed diagnosis of SARS‐CoV‐2 in the follow‐up period, as per the definition of a secondary case in Table 1.

### 2.6 Statistical analysis

The flow of participants through the investigation, including screening, recruitment and loss to follow‐up, was described in an investigation profile. Descriptive analyses were conducted to explore the baseline characteristics of cases and contacts recruited into the investigation. Numbers and proportions are reported for categorical variables, and continuous variables are reported with means and standard deviations or medians with interquartile ranges and minimum and maximum values.

To establish which participants were lost to follow‐up, the availability of laboratory results was used as a proxy to determine if participants completed follow‐up (i.e., they had samples taken on at least two visits where the participant had not died prior to their second visit). The difference between the first and last dates of sampling was calculated—if this was less than or equal to 21 days, the participant was considered completely followed up. If it was more than 21 days, the participant was considered lost to follow‐up.

Key severity parameters include the asymptomatic proportion of cases, the case hospitalisation proportion and the case fatality rate. Transmissibility was characterised by the SAR (with a 95% confidence interval), which was estimated from an unadjusted logistic regression model including all susceptible contacts at baseline (i.e., not seropositive at baseline). The SAR among household contacts was also estimated. Further subgroup analyses to assess transmissibility were not performed because of the limited number of susceptible contacts included in this study.

For all outcomes where there was missing data, it was assumed that those individuals did not experience the event of interest. This approach aligned with the methods implemented during data collection. Data cleaning and analyses were conducted in R version 4.0.0.

## 3 RESULTS

### 3.1 Participant recruitment and classification

**FIGURE 1:**
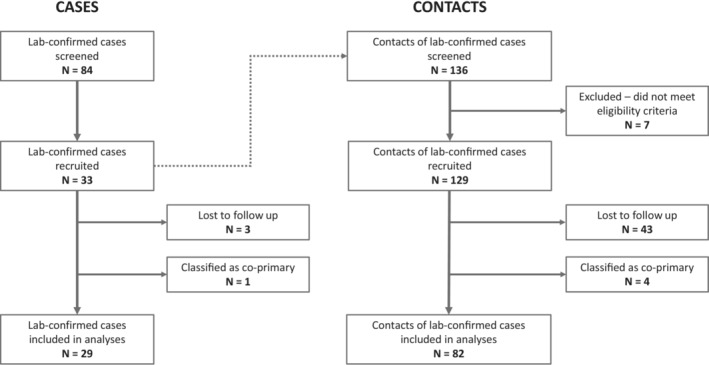
Diagram of participant flow through the investigation.

The first case was recruited on 8 June 2020, and the final follow‐up of participants occurred on 3 December 2020. In total, 33 primary cases were recruited, and 136 close contacts were screened for inclusion. Of these, 129 (94.9%) met the eligibility criteria and were subsequently enrolled in the investigation. Participants were excluded for several reasons, including being unable to be contacted, residing outside of Juba or declining to be involved in the study. A specific reason for exclusion was not recorded for each ineligible individual. Seventeen cases provided at least one household contact, with 27% (35/129) of all contacts classified as household contacts. In total, 46 participants were lost to follow‐up, including three primary cases and 43 contacts (Figure 1). A review of the baseline RT‐PCR results of cases and contacts revealed that there was one index case, which was considered co‐primary with four contacts. These participants were classified as co‐primary and excluded from subsequent analyses. All other (non‐positive) contacts from the co‐primary cases are also excluded, as the chains of transmission are not readily identifiable. Thus, in total, 29 primary cases and 82 contacts were included in the analyses.

### 3.2 Participant characteristics

**FIGURE 2:**
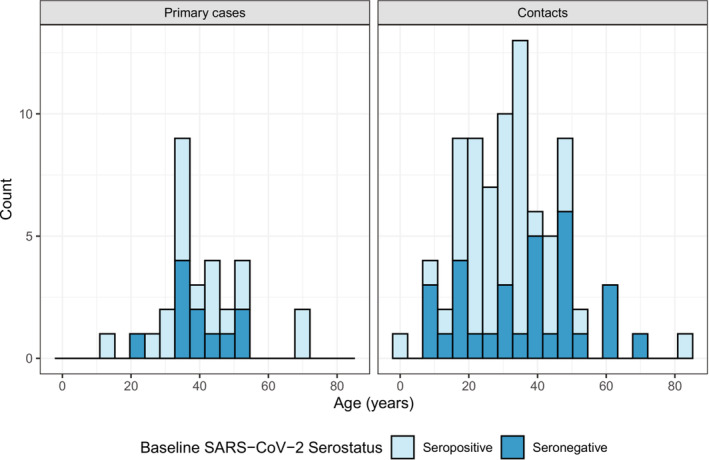
Age distribution of primary cases and contacts by baseline SARS‐CoV‐2 serostatus.

The majority of primary cases (*n* = 23/29, 79.3%) and contacts (*n* = 61/82, 74.4%) were male. By comparison, the proportion of males was lower among participants lost to follow‐up (*n* = 27/46, 58.7%; Appendix A). The median age of primary cases was 38 years (range: 12–72) and of contacts was 32 years (range: 0–83) (Figure 2). The occupation of primary cases varied, with the most common occupations being professionals (*n* = 5/29, 17.2%), businesspeople (*n* = 4/29, 13.8%) or humanitarian/NGO workers (*n* = 4/29, 13.8%). Contacts were most commonly students (*n* = 17/82, 20.7%) and professionals (*n* = 13/82, 15.9%). Three of the 29 cases (10.3%) and no contacts reported having comorbidities (Table 2). Most contacts were exposed to cases in the household (*n* = 45/82, 54.9%) and workplace (*n* = 18/82, 22.0%), while 2/82 (2.4%) listed other exposure locations and 17/82 had missing exposure location data (20.7%). For the duration of the investigation, one contact did not receive any RT‐PCR testing, and one primary case did not participate in serological testing. The majority of both cases (*n* = 18/29, 62.1%) and contacts (*n* = 51/82, 62.2%) were seropositive for SARS‐CoV‐2 at baseline (Figure 2).

**TABLE 2:** Baseline characteristics of participants included in analyses, stratified by primary case and contacts.

Variable	Response	Overall (*N* = 111)	Primary case (*N* = 29)	Contact (*N* = 82)
Sex	Female	27 (24.3%)	6 (20.7%)	21 (25.6%)
	Male	84 (75.7%)	23 (79.3%)	61 (74.4%)
Age, years	Median (IQR) [Range]	35.0 (25.0–45.0) [0–83.0]	38.0 (35.0–46.0) [12.0–72.0]	32.0 (21.2–41.5) [0–83.0]
Occupation	Student	18 (16.2%)	1 (3.4%)	17 (20.7%)
	Professional	18 (16.2%)	5 (17.2%)	13 (15.9%)
	Health care worker	10 (9.0%)	3 (10.3%)	7 (8.5%)
	Humanitarian/NGO	5 (4.5%)	4 (13.8%)	1 (1.2%)
	Religious leader	5 (4.5%)	1 (3.4%)	4 (4.9%)
	Business	4 (3.6%)	4 (13.8%)	0 (0%)
	Farmer	4 (3.6%)	0 (0%)	4 (4.9%)
	Housewife	3 (2.7%)	1 (3.4%)	2 (2.4%)
	Police/Security/Military	3 (2.7%)	3 (10.3%)	0 (0%)
	Service	1 (0.9%)	0 (0%)	1 (1.2%)
	Other	24 (21.6%)	3 (10.3%)	21 (25.6%)
	Unknown or missing	16 (14.4%)	4 (13.8%)	12 (14.7%)
Any comorbidities	Yes^a^	3 (2.7%)	3 (10.3%)	0 (0%)
	No	108 (97.3%)	26 (89.7%)	82 (100%)
Serology was positive for SARS‐CoV‐2 at baseline	Yes	69 (62.2%)	18 (62.1%)	51 (62.2%)
	No	42 (37.8%)	11 (37.9%)	31 (37.8%)

^a^ Of the three primary cases with comorbidities, one had obesity, one had heart disease, and one had heart disease and HIV or another immune deficiency.

### 3.3 Exposures and activities

**FIGURE 3:**
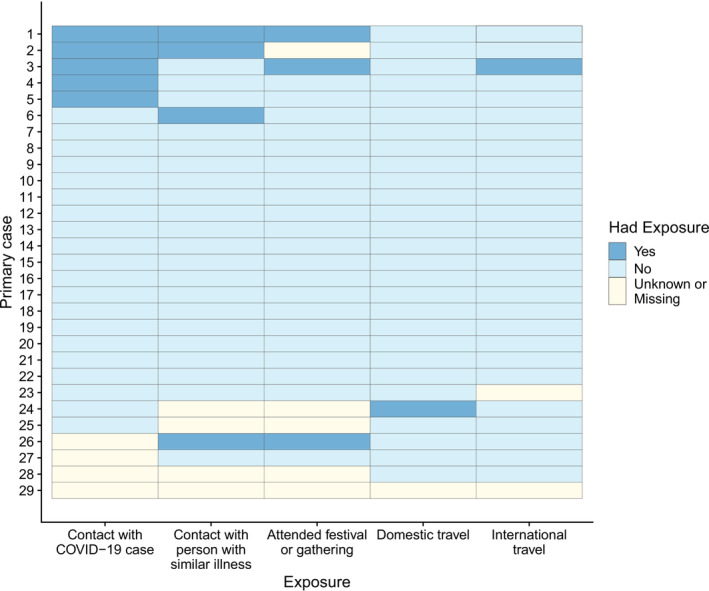
Exposures reported by the 29 primary cases in the 14 days prior to baseline assessment.

Primary cases reported several exposures and activities in the 14 days prior to the baseline assessment that are known risk factors for the acquisition of COVID‐19. These included travel (domestic *n* = 1/29, 3.4%; international *n* = 1/29, 3.4%); attending funerals or mass gatherings (*n* = 3/29, 10.3%); and contact with people with suspected COVID‐19 infection (*n* = 4/29, 13.8%) or a suspected or confirmed COVID‐19 case (*n* = 5/29, 17.2%). Of the 29 primary cases, 8 (27.6%) reported experiencing at least one of the exposures investigated (Figure 3).

### 3.4 Transmission parameters

Among the 82 contacts, most (51/82, 62.2%) were seropositive for SARS‐CoV‐2 at baseline and so were classified as non‐susceptible to COVID‐19 infection. Hence, to reduce bias in the estimate of transmissibility, seropositive contacts were excluded from these analyses. Following exclusions, four secondary cases were identified (two by RT‐PCR and two by seroconversion), linked to 4/29 (13.8%) primary cases. The SAR among the 31 susceptible contacts was 12.9% (4/31, 95% CI: 4.9%, 29.7%). The SAR among susceptible household contacts was 9.1% (1/11, 95% CI: 1.3%, 43.9%).

### 3.5 Clinical manifestations and severity parameters

**FIGURE 4:**
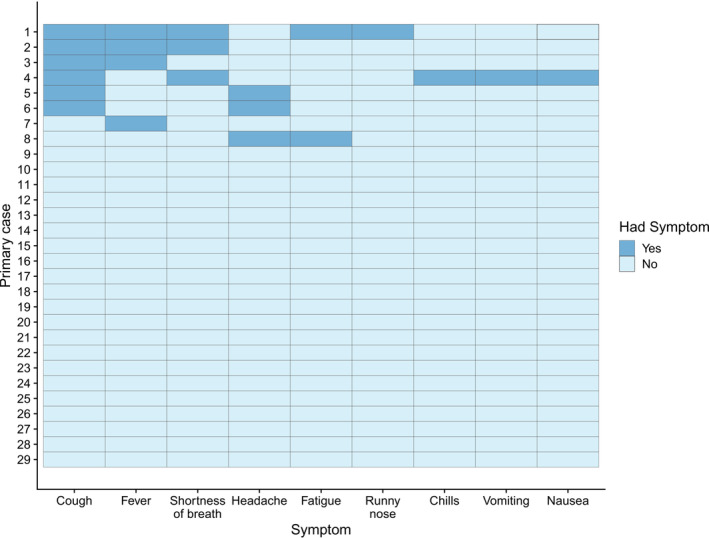
Symptoms recorded among primary cases at baseline.

All (*n* = 9/9, 100.0%) of the secondary cases and the majority (*n* = 21/29, 72.4%) of the primary cases did not report symptoms (*n* = 30/38, 78.9%). Of the eight symptomatic primary cases (27.6%), the most common symptoms reported included coughing (*n* = 6/29, 20.7%), fever or history of fever (*n* = 4/29, 13.8%), shortness of breath (*n* = 3/29, 10.3%) and headache (*n* = 3/29, 10.3%) (Figure 4).

Two primary cases and none of the secondary cases reported visiting an inpatient health facility in the 14 days prior to the baseline assessment. The hospitalisation proportions among primary cases and in total were 6.9% (2/29) and 5.3% (2/38), respectively. Four primary cases (4/29; 13.8%) and no secondary cases reported visiting an outpatient treatment facility in the 14 days prior to the baseline assessment. No cases (primary or secondary) reported visiting traditional medicine practitioners.

One primary case died, with heart failure as the reported cause of death, and COVID‐19 was listed as having an underlying/primary contribution to the death. No deaths were reported among secondary cases (case fatality rate: primary cases 1/29, 3.4%; total 1/38, 2.6%).

## 4 DISCUSSION

This investigation aimed to characterise the transmission and severity of COVID‐19 among cases and their close contacts in Juba, South Sudan. Most cases and contacts were male, and the cohort ages ranged from 0 to 83 years. Most contacts were exposed to cases in the household and workplace. Seroprevalence at baseline was high in the cohort, reducing the pool of susceptible contacts to be considered in transmissibility analyses. Among susceptible contacts, the SAR was 12.9% [95% CI: 4.9%, 29.7%]. Most cases in the cohort did not report symptoms, with cough, fever, headache and shortness of breath being the most commonly reported symptoms. Hospitalisation and death rates (5.3% and 2.6%, respectively) were similar to other studies.^12^, ^13^

The predominance of males in the cohort was a trend seen in other COVID‐19 studies in South Sudan.^6^ One descriptive analysis of 1330 confirmed COVID‐19 cases in the first 60 days of the outbreak in South Sudan reported 77% were male,^7^ despite males comprising 51% of the total South Sudanese population in 2020.^14^ This overrepresentation is expected in an urban population such as Juba, where most respondents were working professionals or students. These groups are mostly male due to male predominance within the education system at all levels,^15^ which leads to higher‐level employment opportunities. Testing rates may have been higher among males than females.^7^ This could be because the vast majority of testing was among travellers,^8^ who are predominantly males travelling for work. It also may be attributed to the marginalisation of women in health services in this setting.^16^

The seroprevalence in the cohort was unexpectedly high at baseline, with 62% of both primary cases and contacts returning a positive SARS‐CoV‐2 serology result. Previous work has shown the median time to antibody seroconversion for SARS‐CoV‐2 is 11 days.^17^ Furthermore, only a minority of primary cases (28%) in the study reported participating in known high‐risk activities and exposures in the previous 14 days, such as international or domestic travel, attending funerals or mass gatherings and being exposed to suspected or confirmed COVID‐19 cases. Considered together, these factors may indicate a delay in the recruitment and baseline assessment of primary cases following infection. This delay could have impacted the detection of secondary cases because many of the contacts may have already been infected at baseline. Moreover, any delay in recruitment may introduce recall bias, impacting the accuracy with which cases report their contacts, exposures and symptoms. The overrepresentation of males in the cohort may also contribute to this higher baseline seroprevalence, as these individuals are at higher risk of infection because of higher levels of participation in education and work‐related travel. Interestingly, only one cluster was identified as having co‐primary infections despite the high baseline seroprevalence among contacts.

There may be other reasons for the high seroprevalence at baseline among the cohort. South Sudan's first COVID‐19 case was reported on 5 April 2020^3^ and recruitment began on 8 June 2020. Given the rapid rate of spread seen elsewhere,^18^ it is possible that there had been substantial infection throughout Juba by the time recruitment started. Policy decisions were rapidly shifting at the start of the pandemic, and tests were not widely available in South Sudan during the early stages of the epidemic. Prior to the commencement of the study, weekly testing peaked at 2423 total tests during the week beginning 25 May 2020, and test positivity peaked at 32.2% in the first week of June 2020.^8^

This investigation reports higher baseline seroprevalence compared with 38.3% (95% CI: 31.8%, 46.5%) reported from a household serosurvey conducted between 10 August and 11 September 2020 in Juba.^19^ The baseline seroprevalence here is also higher than the reported seroprevalence in the African region of 3.0% (95% CI: 1.0%, 9.2%) in April–June 2020.^20^ The differences are likely due to different methodologies—the FFX cohort includes those with confirmed COVID‐19 and their close contacts, that is, those with a higher chance of exposure, and so would be expected to have a higher rate of seropositivity than the general population. The high seroprevalence is also indicative of substantial case under‐ascertainment seen in Africa, with one systematic review estimating that true infections were 100 times larger than those reported.^20^

The SAR (12.9%; 95% CI: 4.9%, 29.7%) was similar to an FFX investigation conducted in neighbouring country of Kenya (15.4%; 95% CI: 10.5%, 22.0%)^21^ but higher than those reported in Rwanda (SAR: 18/615 = 2.9% [1.7%–4.6%]),^22^ Taiwan (SAR 0.7%)^23^ and China (SAR 4.4%).^24^ This difference is likely attributable to the inclusion of more contacts compared with the South Sudan FFX. Contacts in this investigation typically had close connections to each case (i.e., those who lived or worked with the case), likely resulting in a higher SAR. Despite this, higher SAR estimates were also observed in other African countries, including Madagascar (SAR 29.6%; 95% CI: 23.0%, 36.9%)^25^ and Ethiopia (SAR 45.2%; 95% CI: 38.6%, 52.1%).^26^ A recent meta‐analysis highlighted the substantial heterogeneity in SAR estimates obtained from FFX investigations across the globe.^27^ This heterogeneity may be attributed to differences in culture, pandemic activity or implementation of FFX protocols throughout the COVID‐19 pandemic and highlights the need for further standardisation of the FFX investigation protocol.^27^

It is worth noting that most cases in the study did not report symptoms and thus were assumed to be asymptomatic (30/38, 78.9%). This was similar to other findings in South Sudan; one Juba‐based study (*n* = 1330) reported that only 17% of the cohort were symptomatic and 95% of the symptomatic infections were mild.^7^ Compared with several other global FFX studies that were conducted in the early days of the pandemic,^28^, ^29^, ^30^, ^31^, ^32^ symptoms were much less common in the South Sudan FFX. Despite this, the types of symptoms reported were consistent with those in other FFX investigations. Symptoms such as cough, fever and fatigue were widely reported across all regions.^28^, ^30^, ^32^ The case fatality rate from the South Sudan FFX (2.6%; 95% CI: 0.07%, 13.81%) can be contrasted with a cross‐sectional Juba study of 1.1%^7^ and reports from the Bentiu internally displaced population camp in the north of the country, which reported a case fatality rate of 19.1% among confirmed and probable cases and 9.4% among confirmed cases.^6^

The investigation's findings have had important implications for policy and practice in South Sudan. The protocol for follow‐up of cases and contacts was adopted by the Ministry of Health. The detailed clinical and epidemiological findings informed the local COVID‐19 case definition. The South Sudan Ministry of Health implemented this GoData platform for case and contact management as part of the public health response to the COVID‐19 pandemic because of its successful use in this study.

The implementation of a complex FFX investigation in a resource‐limited setting such as South Sudan posed several challenges. Because of limited resources, recruitment was restricted to 25–50 index cases and two to four of their close contacts. Ideally, all close contacts should be approached for enrollment to reduce selection bias in the cohort, but this was not possible in this context because of high rates of participant hesitancy and limited resource availability. Sampling a subset of contacts recruited for each case may provide an incomplete picture of transmission among close contacts. In combination with high baseline seropositivity, this meant that there were far fewer transmission events observed than anticipated. Limited transmission also impacted which analyses were able to be performed. The WHO Unity FFX protocol proposes evaluating the serial interval in addition to the transmissibility and severity parameters reported here.^9^ Given that no secondary cases had symptoms, the serial interval was not able to be estimated as no transmission chains in which both primary and secondary cases were symptomatic were identified.

A key lesson for future investigations is that there was hesitance to participate given the request to draw blood. This hesitation impacted recruitment and could introduce bias as those who were willing to participate may have differed from the population. Reducing serum collection points in future FFX investigations may help improve recruitment rates. An additional limitation was the difficulty in following up with participants. It is important to note that the method for calculating the loss to follow‐up may have overestimated the loss to follow‐up, as participants who attended both visits may not have necessarily had samples taken at each visit, or these samples may have been lost because of lab error. However, this approach is a reasonable proxy for loss to follow‐up, as lab‐confirmed SARS‐CoV‐2 infection is required for the identification of secondary cases and hence for the estimation of the SAR.

Logistical challenges were encountered in the study because of the pandemic and public health measures to control the spread of COVID‐19 (varying degrees of lockdown, global lack of supplies, difficulties with the recruitment of the study team, diverted resources, etc.). These included a delay in obtaining ethical approval as well as limiting staff and resources for the study. This led to delays in the implementation of the study, which, as described above, impacted the timing of the study relative to the introduction of SARS‐CoV‐2 into South Sudan, thus altering the eligible population. Although these challenges are exacerbated within a humanitarian setting, these circumstances are not unique to South Sudan. Reports from high‐resource settings have also cited numerous logistical challenges in implementing an FFX study during the early phases of the COVID‐19 pandemic.^33^ Piloting FFX investigations outside of an outbreak would assist in preparedness for future epidemics or pandemics, allowing for the development of workflows and resource allocation to facilitate easier implementation of FFX investigations when required.^34^

## 5 CONCLUSION

The South Sudan FFX has provided useful insights into the transmission and severity of COVID‐19 in Juba, South Sudan. These findings were used by the South Sudanese Ministry of Health to adapt initial surveillance and contract tracing protocols to the local context. Efforts to standardise FFX protocols should be continued. Improved preparedness for future outbreaks, including integrating FFX investigations into emergency response initiatives, will facilitate a better understanding of the emerging disease and help to inform response priorities.

## AUTHOR CONTRIBUTIONS

**Richard Lino Loro Lako:** Conceptualization; funding acquisition; investigation; methodology; project administration; resources; supervision; writing—review and editing. **Niamh Meagher:** Conceptualization; data curation; formal analysis; methodology; validation; visualization; writing—original draft; writing—review and editing. **Joseph Francis Wamala:** Conceptualization; funding acquisition; investigation; methodology; project administration; resources; supervision; writing—review and editing. **John Ndyahikayo:** Investigation; project administration; writing—review and editing. **Ayesheshem Ademe Tegegne:** Investigation; project administration; writing—review and editing. **Olushayo Oluseun Olu:** Investigation; project administration; writing—review and editing. **David J Price:** Formal analysis; supervision; visualization; writing—original draft; writing—review and editing. **Soatiana Rajatonirina:** Supervision; writing—review and editing. **Elise Farley:** Project administration; supervision; writing—original draft; writing—review and editing. **Joseph Chukwudi Okeibunor:** Project administration; supervision; writing—review and editing. **Valerie Ann Mize:** Data curation; investigation; methodology; project administration; supervision; writing—review and editing.

## CONFLICT OF INTEREST STATEMENT

The authors declare no conflicts of interest.

### PEER REVIEW

The peer review history for this article is available at https://www.webofscience.com/api/gateway/wos/peer-review/10.1111/irv.13200.

## ETHICS APPROVAL

Ethical approval was obtained from the South Sudan Ministry of Health Research and Ethical Review Board (MOH/ERB/04/2020). Written informed consent was obtained from all literate respondents; illiterate respondents provided a thumbprint and a signature from a literate witness. Consent for children to participate in the investigation was obtained from their parents or caretakers, and assent was secured for children 12 years and above as a requirement for securing participation. Participants' privacy and confidentiality were ensured. Key information pertaining to individual participants was kept confidential. Codes were provided instead of names when submitting samples for laboratory analysis.

## Data Availability

The summary data that support the findings of this study are openly available on Zenodo at https://zenodo.org/record/6131555#.ZB-OyXZByUk under the title ‘South Sudan FFX Unity study for COVID‐19’. Line‐listed data is not publicly available to preserve the privacy of participants and to comply with ethical considerations.

## APPENDIX A

**Table:** Baseline characteristics of participants subsequently lost to follow up, overall and for primary cases and contacts.

Variable	Response	Overall (*N* = 46)	Primary case (*N* = 3)	Contact (*N* = 43)
Sex	Female	19 (41.3%)	1 (33.3%)	18 (41.9%)
	Male	27 (58.7%)	2 (66.7%)	25 (58.1%)
Age, years	Median (IQR) [Range]	31.0 (24.0, 36.8) [0, 91.0]	47.0 (39.5, 57.0) [32.0–67.0]	30.0 (24.0, 36.0) [0, 91.0]
Occupation	Student	12 (26.1%)	0 (0.0%)	12 (27.9%)
	Professional	4 (8.7%)	1 (33.3%)	3 (7.0%)
	Health care worker	2 (4.3%)	0 (0.0%)	2 (4.7%)
	Business	2 (4.3%)	0 (0.0%)	2 (4.7%)
	Housewife	2 (4.3%)	0 (0.0%)	2 (4.7%)
	Police/Security/Military	2 (4.3%)	0 (0.0%)	2 (4.7%)
	Other	9 (19.6%)	1 (33.3%)	8 (18.6%)
	Unknown or missing	13 (28.3%)	1 (33.3%)	12 (27.9%)
Any comorbidities	Yes	0 (0.0%)	0 (0.0%)	0 (0%)
	No	46 (100.0%)	3 (100.0%)	43 (100.0%)
Serology was positive for SARS‐CoV‐2 at baseline	Yes	29 (63.0%)	2 (66.7%)	27 (62.8%)
	No	17 (37.0%)	1 (33.3%)	16 (37.2%)
